# Noninvasive detection of elevated ICP using spontaneous tympanic membrane pulsation

**DOI:** 10.1038/s41598-021-01079-8

**Published:** 2021-11-09

**Authors:** Rajkumar Dhar, Richard H. Sandler, Kim Manwaring, Nathan Kostick, Hansen A. Mansy

**Affiliations:** 1 Biomedical Acoustics Research Laboratory, University of Central Florida, Orlando, FL 32816 USA; 2 Pediatric Neurosurgery, Arnold Palmer Children’s Hospital, Orlando, FL 32806 USA; 3 College of Medicine, University of Central Florida, Orlando, FL 32827 USA

**Keywords:** Diagnosis, Biomedical engineering, Mechanical engineering, Neuroscience, Neurological disorders

## Abstract

Neurological conditions such as traumatic brain injury (TBI) and hydrocephalus may lead to intracranial pressure (ICP) elevation. Current diagnosis methods rely on direct pressure measurement, while CT, MRI and other expensive imaging may be used. However, these invasive or expensive testing methods are often delayed because symptoms of elevated ICP are non-specific. Invasive methods, such as intraventricular catheter, subdural screw, epidural sensor, lumbar puncture, are associated with an increased risk of infection and hemorrhage. On the other hand, noninvasive, low-cost, accurate methods of ICP monitoring can help avoid risks and reduce costs while expediting diagnosis and treatment. The current study proposes and evaluates a novel method for noninvasive ICP monitoring using tympanic membrane pulsation (TMp). These signals are believed to be transmitted from ICP to the auditory system through the cochlear aqueduct. Fifteen healthy subjects were recruited and TMp signals were acquired noninvasively while the subjects performed maneuvers that are known to change ICP. A custom made system utilizing a stethoscope headset and a pressure transducer was used to perform these measurements. Maneuvers included head-up-tilt, head-down-tilt and hyperventilation. When elevated ICP was induced, significant TMp waveform morphological changes were observed in each subject (*p* < 0.01). These changes include certain waveform slopes and high frequency wave features. The observed changes were reversed by the maneuvers that decreased ICP (*p* < .01). The study results suggest that TMp waveform measurement and analysis may offer an inexpensive, noninvasive, accurate tool for detection and monitoring of ICP elevations. Further studies are warranted to validate this technique in patients with pathologically elevated ICP.

## Introduction

Intracranial pressure is the pressure in the cerebrospinal fluid (CSF) that surrounds the brain tissue and spinal cord^1^. The normal range of ICP in healthy adults is 5–15 mmHg^1^. Neurological conditions such as hydrocephalus and traumatic brain injury (TBI) can lead to intracranial pressure (ICP) elevation, which is associated with serious complications, including severe headaches, blurred vision, problems in moving or talking, seizures and even death. Due to these potential risks, prompt medical attention and reliable monitoring may be needed for patients with elevated ICP. Current diagnosis relies on direct pressure measurement, while computer tomography scan, magnetic resonance imaging (MRI) and other expensive imaging can also be used. However, these invasive or expensive tests are often delayed because the telltale symptoms mentioned above are non-specific, and easily attributed to viral or other common benign maladies. Although exceeding the normal range is considered elevated ICP, not all ICP elevations require treatment, and treatment strategy is guided by the etiology. For example, in the case of TBI, which caused 61,000 deaths in the USA in 2017^2^, ICP > 20 mmHg indicates medical intervention^3^. The primary brain injury in TBI may be followed by a series of secondary events that lead to further deterioration of the patient’s clinical status^4^. One of the key signs of secondary injuries is the rise of ICP^4^. Other conditions associated with elevated ICP include hydrocephalus, intracranial hemorrhage and meningitis^5^. Elevated ICP can also lead to brain herniation and tissue ischemia. Accurate and rapid tools for monitoring ICP can lead to early detection of elevated ICP, which allows for aggressive treatment and avoidance of devastating consequences. Current direct methods of ICP monitoring include intraventricular catheter, subdural screw, epidural sensor, lumbar puncture, all of which are invasive and associated with complications, such as risk of infection, malposition, and hemorrhage^6^. Noninvasive methods of ICP monitoring help avoid these risks while expediting diagnosis and treatment.

**Figure 1:**
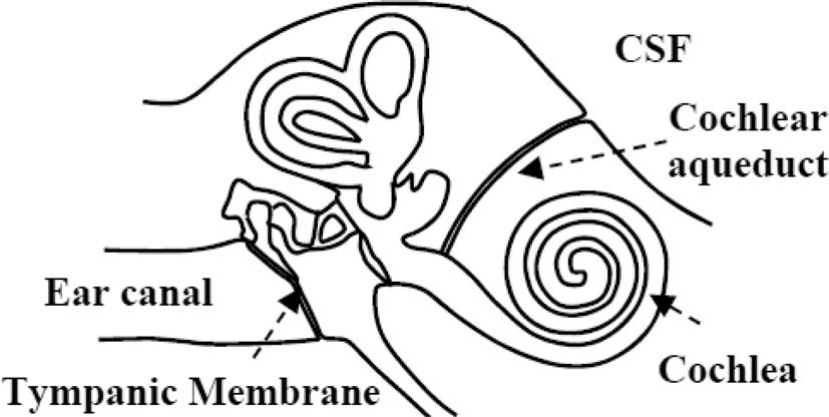
Transmission of ICP pulsation from CSF space to tympanic membrane.

Many noninvasive ICP monitoring techniques have been studied^7^, including optic nerve sheath diameter measurement^8^, MRI^9^, transcranial doppler ultrasound^10^, and tympanic membrane (TM) displacement^11^. However, to date, none of these techniques have been proven to be reliable and, new methods are still needed. This study focuses on the use of tympanic membrane pulsation (TMp) for ICP monitoring. This technique relies on the possible connection between the subarachnoid space of the brain and the perilymphatic space of the cochlea. An animal study suggested that ICP pulsations may be transmitted via CSF to the inner ear through the cochlear aqueduct, which connects subarachnoid and perilymphatic spaces^12^ (Fig. 1). The exact transmission path in humans is not known, but it may be similar. The transmitted pressure wave then may propagate from the inner ear to the ossicles (malleus, incus, and stapes) in the middle ear to the TM^13^.

ICP waveform is known to undergo distinctive morphological changes with ICP elevation^15^. Therefore, several studies suggested that analysis of ICP waveforms may serve as a clinical tool to monitor intracranial compliance^15–18^. However, direct measurement of the ICP waveform is invasive, which limits its utility. Some studies demonstrated the correlation between ICP levels and tympanic membrane displacement (TMD) which is measured as the ear canal volume changes as a function of time^19–21^. TMD can be thought of as “evoked” or “spontaneous”, where evoked TMD is defined as the mean TM volume displacement, and spontaneous TMD is the time-dependent TM movement waveform^22^. However, several studies suggested that there is large inter-subject variability in the evoked TMD measurements, which has made it difficult to implement as a noninvasive method for ICP monitoring^23,24^. On the other hand, studies suggested that ICP oscillations can be transmitted to the tympanic membrane and spontaneous TMD waveform^19,22^. Recent studies recommended that spontaneous tympanic membrane movements may be also acquired using sensitive pressure transducers connected to the ear canal with an airtight tubing^25–27^. The current study presents additional information about the latter approach.

## Materials and methods

**Figure 2:**
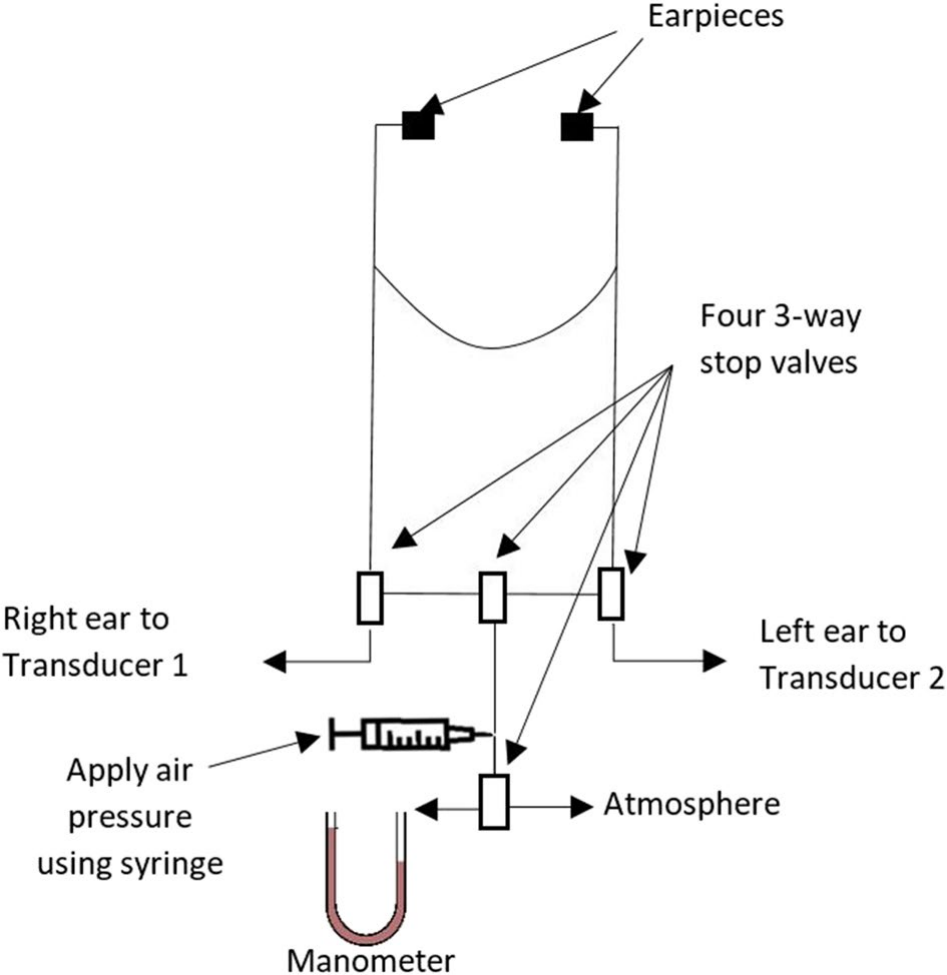
Stethoscope setup for acquiring the TMp signal from the right and left ears. 3-way stop valves are used to route the TMp signal to two pressure transducers where the signal from each ear is connected to one transducer and is kept separate. Alternately, the valves can mix the two signals and route them to the same transducer. The middle stop valves were used to vent the system to atmosphere and to test the system air tightness. Tightness was tested by applying about 2 cm of water pressure using a syringe.

Two low-pressure variable-reluctance pressure sensors (DP103, diaphragm dash number 10, Validyne Engineering, Los Angeles, CA 91324, USA) were used to acquire TMp vibration. A double-tubing stethoscope (Sprague rappaport stethoscope, ESR-112, Elite Medical Instrument Inc., Fullerton, CA 92831, USA) was connected to a hub of four three-way stop valves (Fig. 2). The middle valves were used to test the air tightness of the system by connecting it to a manometer and also to vent the pressure out of the system between data acquisition segments (as described below). By controlling the top 3 stop valves, TMp from both ears can be combined and acquired as one signal or separated and acquired independently by a different pressure transducer. The pressure transducers were mounted on an IV pole similar to clinical settings. Flexible PVC tubes connected pressure transducers and stop valves and were 6 feet long (diameter = 5 mm).

### Subject selection

15 subjects (age 23–34 years) were recruited for the study after the experimental protocol was approved by University of Central Florida Institutional Review Board. The relatively young population may be preferred as studies have suggested that cochlear aqueduct patency decreases with age^28^, which could potentially reduce signal quality. Future studies would consider a larger population with a wider age range.

### Data acquisition

**Figure 3:**
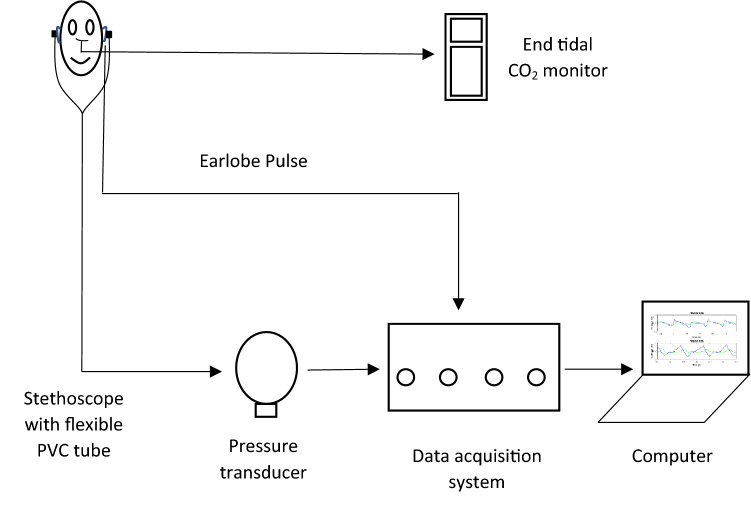
Experimental setup.

After obtaining informed consents from the subjects, stethoscope earpieces were securely placed in their ears to acquire the TMp signals. A lubricant (Oto-ease Earmold Lubricant, Westone Laboratories, Colorado Springs, CO 80906, USA) was rubbed on the earpieces before the experiment to ensure a proper seal between the earpieces and the external ear canal. An optical ear lobe pulse sensor (Sparkfun Electronics, Niwot, CO, USA) was clipped to the subject’s earlobe to monitor the ear lobe pulse. A nasal cannula was worn by the subject. The nasal cannula was connected to an end-Tidal CO_2_ (ETCO_2_) sensor (MicroCap9, MDPro, San Diego, CA 92117). The outputs from pressure sensors and earlobe pulse sensor were simultaneously acquired by an iWorx data acquisition system (IX-TA-220, iWorx Systems Inc., Dover, NH 03820, USA) and software^29^ that allowed real-time monitoring of signals. To preserve time- and frequency-domain information, a sampling frequency of 2000 Hz was used. The experiment setup is shown in Fig. 3.

The air tightness of the whole system was checked at the beginning of each data acquisition session. Here, the tubing from the earpieces, the transducer, and the manometer were all connected together at the hub by properly adjusting all the valves. The venting to atmosphere port was closed and a small pressure (2–3 cm of water) was applied to the system by the syringe. Air tightness was confirmed when this pressure was maintained for one minute. If an air leak was detected, the manometer could be connected to each air space (left or right) separately to isolate the leak source. No leak was detected in any of the study subjects, suggesting the robustness of this system. Once the leak checking was done, the system was vented to the atmosphere by opening the venting valve then closing it once the manometer indicated zero pressure.

**Figure 4:**
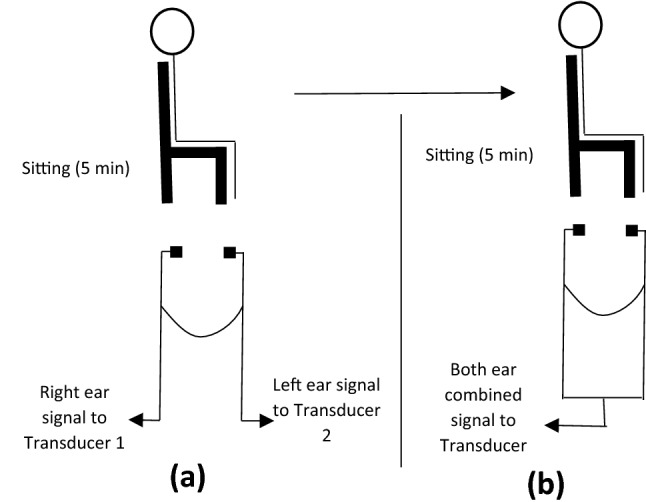
Schematic representation of the TMp measurement methodology with the subject in the sitting position (baseline). The TMp signal was measured in **(a)** each ear separately and **(b)** both ears collectively to identify the signal with highest amplitude for use in the rest of the study, i.e., tilt table experiments.

At the beginning of each trial, valves were set where each transducer was connected to one ear only to record TMp signals from the two ears separately (Fig. 4a). Subjects were asked to refrain from any movement to help minimize noise in the measured TMp. Baseline TMp and earlobe pulse were recorded for five minutes in the sitting position. Then, valves were set to acquire TMp signals from both ears collectively by a single transducer (Fig. 4b). The system was again vented to the atmosphere and, then sealed before data was collected for another 5 min. Baseline data was used to select the optimal (i.e., highest signal quality, see Eq. 1) configuration to be used during testing.

**Figure 5:**
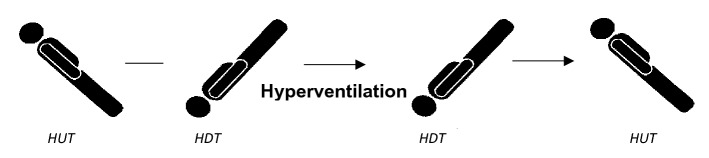
Representation of maneuvers used to raise and reduce ICP in healthy subjects.

To investigate the effect of ICP changes on the TMp waveform, data was recorded at different tilt angles and with and without hyperventilation (Fig. 5). Here, subjects were asked to move to the tilt table and rest at 45° head-up-tilt (HUT) position. Subjects were secured to the table using shoulder straps after adjusting the footrest of the table to match subject height. Feet were kept secured by sliding them between the table’s foam leg roller and the feet holder.

After two minutes of rest, the system was vented to the atmosphere and then sealed again. The signal output from pressure transducer(s) and earlobe pulse sensor and partial pressure reading (in mmHg) from the ETCO_2_ sensor were recorded at 45° tilt table position for 1 min. Then the table was tilted to the head-down-tilt (HDT) position (at -45° to -30° depending on the subject’s comfort) to acquire data at the elevated ICP state. Then, data was acquired for 30 s after 5 s of stabilization time. This was followed by subject hyperventilation while continuously monitoring the ETCO_2_ sensor reading. When the partial pressure reading dropped by 15–20 mmHg from the initial value (typically after 10–30 s), subjects were asked to stop hyperventilating and data was recorded for another 30 s following 5 s of stabilization time at the same position. Then subjects were tilted back to 45° head-up position and, after stabilization for 10–15 s, 1 min of data was acquired from the pressure, earlobe pulse sensor and ETCO_2_ sensor. All the procedures were performed following relevant regulations and guidelines.

### Signal processing and segmentation

The TMp signal preprocessing code was written using a commercial software package (Matlab, Mathworks, Natick, MA)^30^. The raw TMp signal was filtered to remove environmental, electronic and respiratory noises. ICP waveforms are known to have most of their energy below the hearing threshold of 20 Hz^31^ and hence, higher frequencies would mostly contain noise. The cochlear aqueduct, which links between ICP and TM pulsations, also acts as a low-pass filter to filter out cardiac and respiration induced pulses and frequencies above 20 Hz^32^. Therefore, a 4th order band pass filter with a passband of 1 to 20 Hz was applied.

To segment TMp cycles, the earlobe pulse was used as a reference signal since it typically shows relatively repeatable waveforms with a clear positive peak. Matlab function ‘findpeaks’ was used to locate the peaks of the earlobe pulse signal as proposed in a previous study^33^. TMp events were identified by choosing a search interval of 0.7 s before and after the earlobe pulse peaks. Minima in the search intervals were taken as the beginning and end of each TMp event, with each event corresponding to an earlobe pulse. Individual TMp events were aligned in time and were averaged to reduce noise. The mean TMp waveform was used to represent the TMp signal and extract signal features that may correlate with ICP changes.

## Results

**Figure 6:**
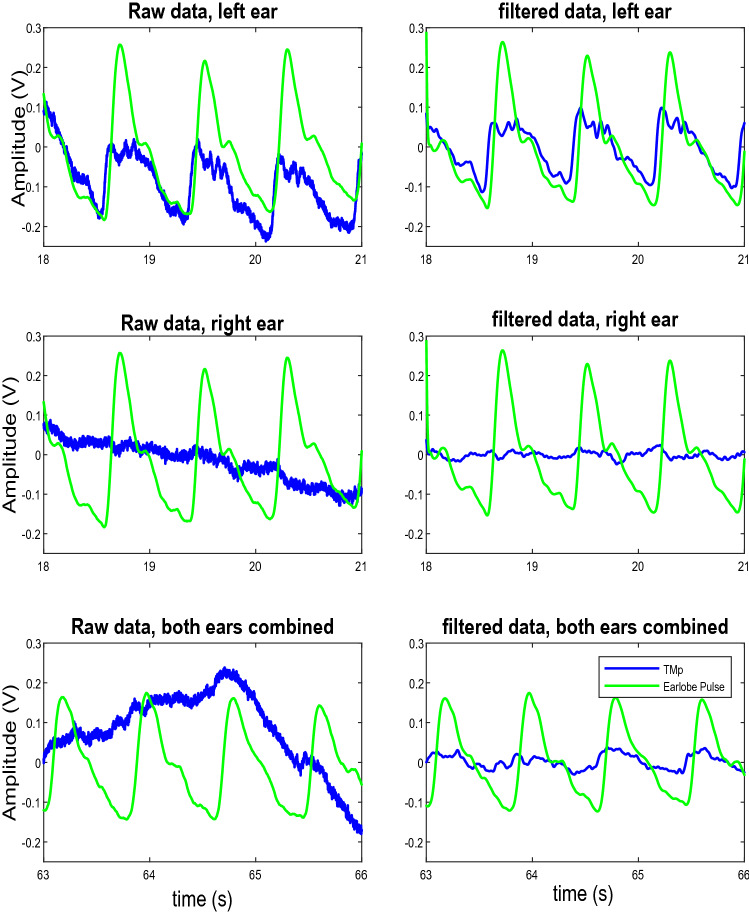
Raw (left column) and filtered (right column) TMp signals from left (top row), right (middle row) and combined both (bottom row) ears for subject 4. The TMp signal is blue while with earlobe pulse is green.

Ideally, the TMp waveform from both ears would be a superimposition of the TMp waveforms from the left and right ears when measured separately. This was observed in most subjects. For example, Fig. 6 shows the recorded signals from the left, right and both-ears-combined signals of one of the subjects in the sitting (i.e., baseline) position.

**Figure 7:**
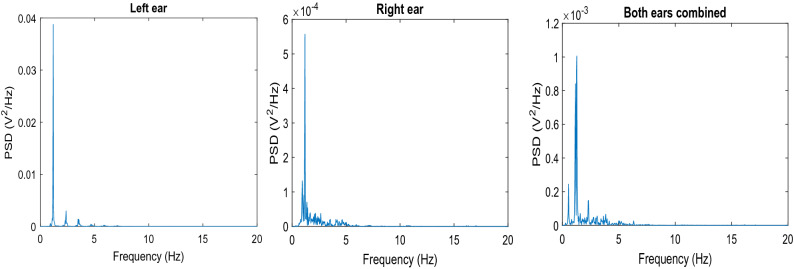
PSD of the filtered TMp signals of Fig. 6.

The left column shows the raw signals (TMp and earlobe pulse, blue and green lines, respectively) and the right column shows the filtered signals. The top, middle and bottom rows are the left, right and both-ears-combined signals respectively, with the corresponding earlobe pulses. This figure suggests that in this subject, the left ear TMp signal is likely a better candidate for tilt test than the other two because of its waveform periodicity and multiple clear peaks. Figure 7 shows the power spectral density (PSD) of the three filtered signals shown in Fig. 6. The quality of TMp signals was quantified using Eq. (1) and the PSD of the filtered TMp signals.

1 $$Signal \, quality=\frac{maximum \, PSD \, of \, TMp \, signal}{Total \, sum \, of \, PSD \, of \, TMp \, signal}$$

Table 1 shows the signal quality of right, left and combined-both-ears TMp signals at baseline (sitting position) for all study subjects. The signal with the best quality for each subject was chosen during tilt table testing where ICP was varied. For example, in the case of subject 4 (waveforms shown in Fig. 6), the TMp signal from the left ear had noticeably higher quality and, therefore, the left ear was selected to acquire the TMp signal during tilt table testing for this subject.

**Table 1:** Signal quality of TMp signals from right, left and both ears for all the subjects.

Subject index	Right ear	Left ear	Both ears
1	0.25	0.28	0.30
2	0.30	0.29	0.33
3	0.12	0.17	0.23
4	0.17	0.37	0.13
5	0.3	0.31	0.37
6	0.27	0.25	0.32
7	0.33	0.40	0.43
8	0.28	0.32	0.35
9	0.31	0.33	0.37
10	0.13	0.30	0.11
11	0.15	0.38	0.31
12	0.26	0.25	0.32
13	0.32	0.38	0.36
14	0.40	0.11	0.25
15	0.25	0.33	0.22

**Figure 8:**
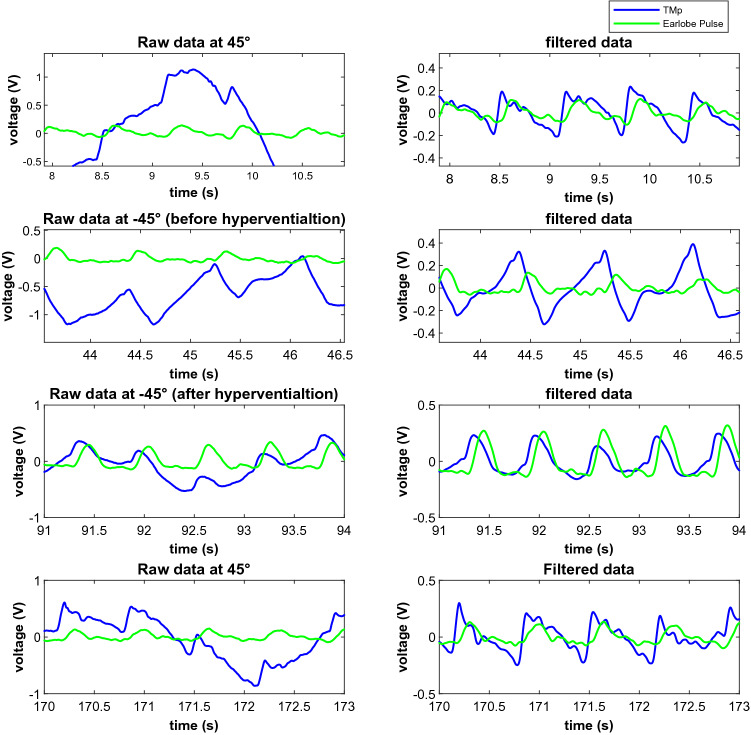
Raw (left column) and filtered (right column) TMp and Earlobe pulse signals (blue and green lines, respectively) from left ear of a representative subject (Subject 2) at different tilt angle positions. 1st row: head up, 2nd row: head down, 3rd row: head down after hyperventilation, 4th row: back to the head up position.

**Figure 9:**
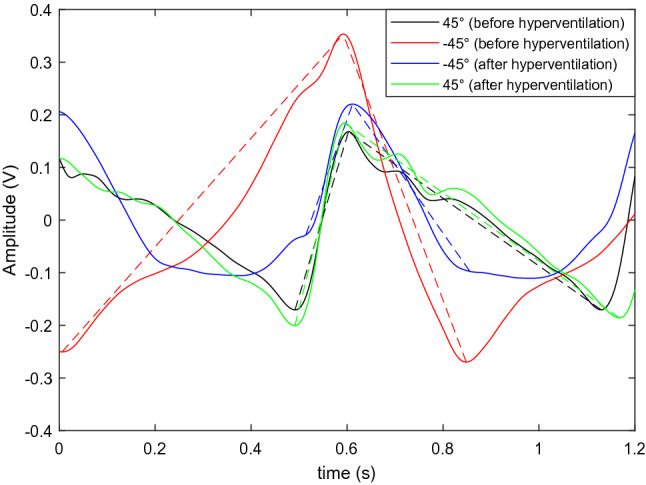
The average TMp waveforms and associated slopes of Subject 2 at different tilt angles and hyperventilation. Black lines represent TMp waveform at 45° head up position at the beginning of the data acquisition. TMp waveform changes its pattern at head down tilt angle shown by red lines. After hyperventilation which reduces ICP, waveform shape again changes as seen by blue lines. After returning the subject to 45° head up position, waveform (green lines) attains the shape which is very close to the initial TMp waveform. Solid lines are the TMp waveforms while the dotted lines show the attack and decay rates (i.e., signal slopes before and after the peak).

Figure 8 shows the raw and filtered TMp and earlobe pulse signals at different tilt angles and for hyperventilation of a representative subject (Subject 2). Rows 1 through 4 show signals for: the initial 45° head-up position, −45° head down position, −45° after hyperventilation, and return to the 45° head-up position, respectively. These results suggest noticeable TMp waveform changes with subject tilting and with hyperventilation. Figure 9 shows the average TMp signals at different tilt angles of the same subject. At the head-down position, the waveform showed a steeper slope after the peak compared to the head-up position. After hyperventilation, the waveform tended to shift towards the head up position shape. After returning to the 45° tilt angle, the TMp waveform regained its initial shape, suggesting effect reversibility and high intra-session repeatability. The straight dotted lines superimposed over the waveforms were used to calculate the attack and decay rates of TMp (i.e., the slope of the wave before and after the peak).

**Figure 10:**
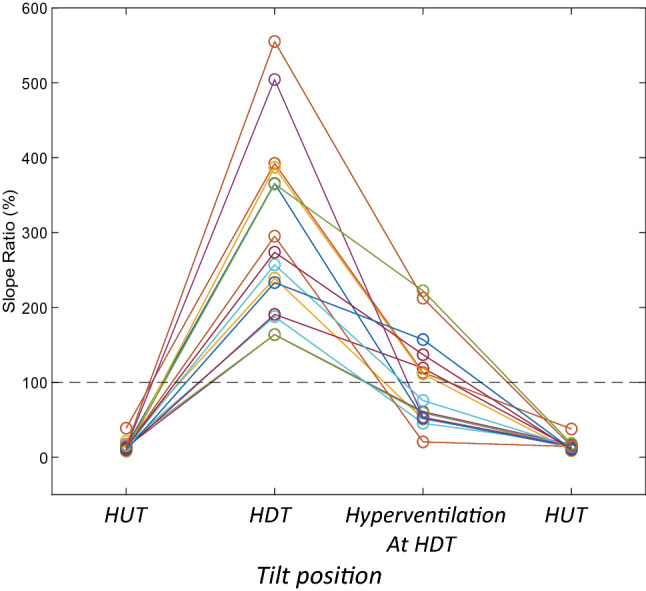
The slope ratios (in percentage) of TMp waveforms after and before the peak for all the 15 subjects at different tilt angles. The ratios start at low values before rising rapidly with tilt induced ICP increase. Hyperventilation tended to reverse tilt effect. The slope ratio was significantly different between normal and elevated ICP states (P = 2.3E−07, unpaired two tailed t-test). A threshold (dashed line) can separate the normal and elevated ICP with 100% accuracy.

To quantitatively compare the waveform change, the ratio of slopes before and after the peak of mean TMp signals at each state was calculated using Eq. (2). These slope ratios at different tilt angles and hyperventilation states were shown in Fig. 10 for all subjects. This slope ratio was significantly different between normal and elevated ICP states (P = 2.3E−07, unpaired two tailed t-test). A threshold (dashed line) can separate normal and elevated ICP in all study subjects. Separation accuracy can be calculated using Eq. (3) and was 100% in the study subjects.

2 $$slope \, ratio=-\frac{\mathrm{slope } \, \mathrm{of} \, \mathrm{ TMp} \, \mathrm{signal} \, \mathrm{after} \, \mathrm{TMp} \, \mathrm{peak}}{\mathrm{slope} \, \mathrm{ of} \, \mathrm{TMp} \, \mathrm{signal} \, \mathrm{before} \, \mathrm{TMp} \, \mathrm{peak }} \times 100$$

3 $$Accuracy=\frac{\mathrm{True} \, \mathrm{Positive}+\mathrm{ True} \, \mathrm{Negative}}{\mathrm{True} \, \mathrm{Positive}+\mathrm{ True} \, \mathrm{Negative}+\mathrm{Flase} \, \mathrm{Positive}+\mathrm{ False} \, \mathrm{Negative }}$$

**Figure 11:**
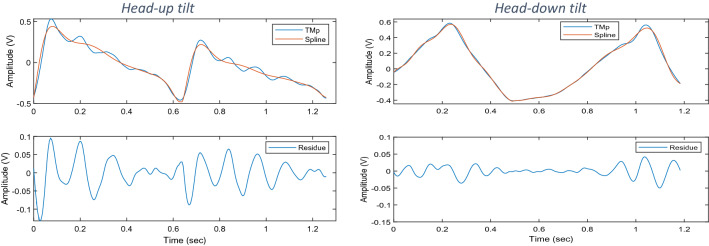
A representative TMp and fitted spline signals (top row) and the residue signal (bottom row) at head-up-tilt (left column) and head-down-tilt (right column) positions. The residue signal equals the difference between TMp and spline.

This study found that the TMp waveform at the head-up-tilt position has morphological similarity to the ICP waveform, both having distinctive higher-frequency peaks on the downstroke (see Fig. 11, left column). Although at the head-down-tilt, these peaks seem to diminish (right column of Fig. 11). To get a quantitative measure of this effect, smooth splines were fitted to TMp waveforms (after normalization by peak-to-peak values). Then the splines were subtracted from their original TMp signals. The subtracted signals, which were named “residue” signals, would mostly contain the higher frequency peaks of the TMp signals (Fig. 11, bottom row). These peaks appeared to have frequencies above the 2nd harmonic of the heart rate.

To estimate the relative strength of these peaks, the PSD of the TMp and their respective residue signals were calculated. The energy ratio in these two signals above the 2nd harmonic of the heart rate was then computed for both HUT and HDT positions using Eq. (4). The energy ratio indicates the relative strength of the high frequency peaks of the TMp signal.

4 $$Energy \, ratio=\frac{Energy \, of \, the \, residue \, signal \, above \, the \, 2nd \, harmonic}{Energy \, of \, the \, of \, the \, TMp \, signal \, above \, the \, 2nd \, harmonic}$$

**Figure 12:**
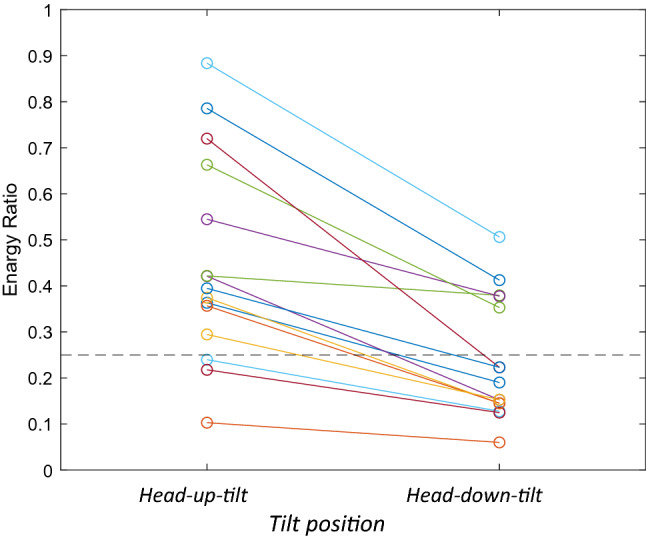
The energy ratios of TMp waveforms at the head-up-tilt and head-down-tilt for all 15 subjects. The ratios were significantly lower (P = .004, unpaired two tailed t-test) for the HDT. A threshold (dashed line) at 0.25 separates the normal and elevated ICP with 76.67% accuracy. A significant ratio reduction with HDT was observed in all study subjects as seen in Table 2 (P = 4.3E−9, paired two tailed t-test) Therefore, if a HUT position energy ratio is available, the accuracy of detecting elevated ICP (HDT) will rise to 100%.

The energy ratios for the normal and elevated ICP states (HUT and HDT, respectively) are shown in Fig. 12 for all subjects. This ratio was significantly different between normal and elevated ICP states (P = 0.004, unpaired t-test). A threshold (dashed line) can separate normal and elevated ICP with 76.67% accuracy. Accuracy was calculated using Eq. (3). The data in Table 2 shows a consistent relative decrease in energy ratio with HDT in all study subjects that was significant (P = 4.3E−9, paired two tailed t-test).

**Table 2:** shows this energy ratio at HUT and HDT.

Subject index	HUT	HDT	Percentage reduction
1	0.363	0.190	47.7
2	0.103	0.060	41.7
3	0.374	0.145	61.3
4	0.421	0.152	63.8
5	0.422	0.379	10.1
6	0.234	0.128	46.7
7	0.218	0.125	42.7
8	0.786	0.412	47.5
9	0.357	0.145	59.3
10	0.294	0.153	48.1
11	0.545	0.378	30.7
12	0.663	0.353	46.8
13	0.885	0.506	42.7
14	0.719	0.223	69.0
15	0.395	0.223	43.5

Noticeable percentage reduction in ratio can be seen at HDT compared with to HUT in all the subjects.

## Discussion

Previous studies attempted to extract ICP waveform features that may be useful for managing neurologic disorders like hydrocephalus, TBI, intracerebral hemorrhage and subarachnoid hemorrhage^34–36^. Studies also suggested that these features may be better markers for predicting pathological outcomes than mean ICP^37–39^. The mean ICP is a static quantity, while the ICP waveform contains additional time and frequency information that may have diagnostic value. In addition, the ICP waveform is a dynamic signal that may be easier to transmit through the cochlear aqueduct and the auditory system and can be detected noninvasively as tympanic membrane movements.

This study investigated a new noninvasive method for elevated ICP detection using tympanic membrane pulsation waveform changes. Healthy subjects performed various physiological maneuvers known to modulate ICP. Maneuvers included head-down-tilt and hyperventilation. Head-down-tilt instantaneously increases ICP due to fluid shift to the upper body^40,41^ while short term hyperventilation is known to cause cerebral vasoconstriction and to quickly lower cerebral blood flow and ICP^42^.

**Figure 13:**
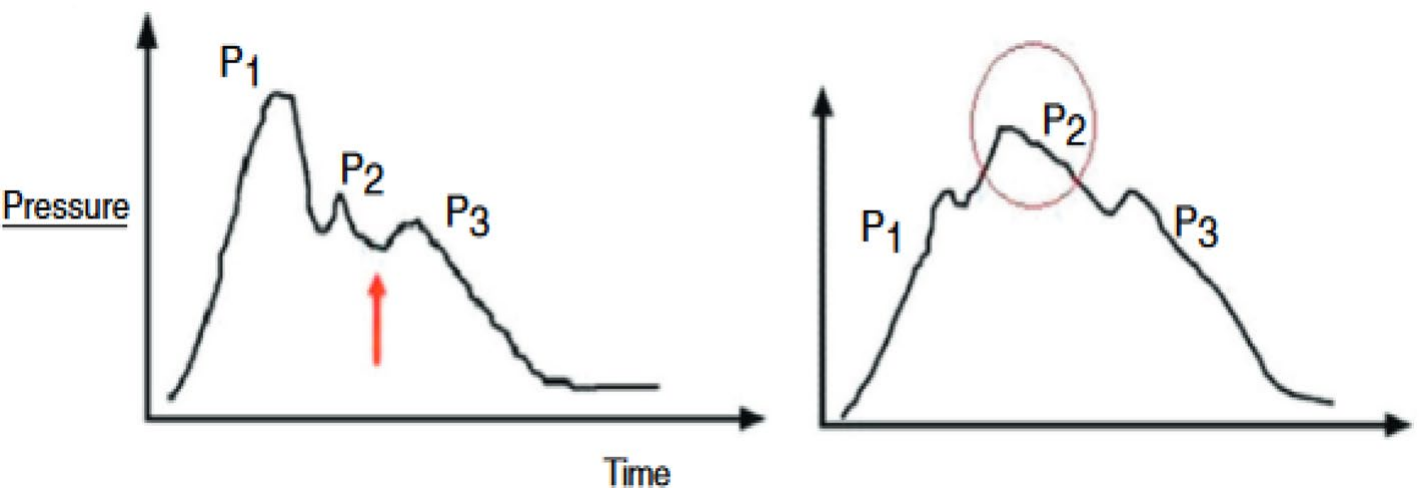
ICP waveform: (Left) non-pathological recording of ICP waveform showing three distinctive peaks (P1, P2 and P3), the orange arrow marks dicrotic notch; (right) prominent P2 which indicates intracranial noncompliance. Figure is adapted from^42^.

To understand the TMp waveform changes seen in the current study, a discussion of the ICP waveform morphology would be helpful. As shown in Fig. 13, ICP waveform has three distinctive peaks: P1 (percussion wave), P2 (tidal wave) and P3 (dicrotic wave). P1 may originate from arterial pulsation transmitting to choroid plexus, P2 is believed to result from arterial pulsation rebounded from brain parenchyma (thus affected by cerebral compliance) and P3 may represent aortic valve closure^18,43,44^. For normal ICP, P1 remains the highest peak. As ICP increases P2 may become more prominent (exceeding P1) and may indicate lower intracranial compliance^43^. This increase in P2 amplitude may affect ICP waveform attack and decay rates.

Further, a previous study on the origin of ICP pulsation discussed the contribution of arterial blood pressure (ABP) and cerebral arterial blood volume to the ICP waveform^45^. The study suggested that P1 in the ICP waveform is associated with the arterial pressure systole. Another study found that there is large spectral coherence between both ABP and ICP and spontaneous tympanic membrane pulsation waveforms^19^. This may explain the TMp waveform trends seen in the current study, where waveform slopes and peaks changed as ICP was varied (see for example, Figs. 9 and 11).

The results of the current study showed that at the head-down-tilt (i.e., increased ICP), certain TMp waveform peaks diminished, which is consistent with the findings by Stettin et al.^46^. In addition, Hommerich^47^ concluded that the tympanic membrane’s ability to vibrate decreases with increased ICP. This may further explain the decreased amplitude of TMp peaks (Fig. 11) and the inhibition of high frequency energy (Table 2) observed in the current study. This signal feature (i.e., the energy ratio in Table 2) had a detection accuracy of 100% and 76% in the current study when its baseline value was available or not available, respectively. It is to be noted that relying on amplitude change or energy change relative to a baseline value would require the availability of a TMp baseline measurement, which may not always be possible and would limit utility.

Another marked difference between HUT and HDT waveforms was the reduced upstroke slope and increased decline slope with HDT, which resulted in the observed changes in the slope ratios (P = 2.3E-07, unpaired two tailed t-test, Fig. 10). It is important to note that the slope ratio (of Eq. 2) does not suffer from the limitation of relying on baseline measurements and, therefore, may find wider clinical use for elevated ICP detection, especially in cases when a baseline value is not available.

The slope changes seen in the current study may be explained by the possible stiffening of the brain due to elevated ICP^48^. Here, as ICP increases at the head-down-tilt position, cerebral blood flow decreases due to compression of blood vessels^49^ and potential more prominence of the P2 wave^43^. This phenomenon may have resulted in a reduced upstroke ICP slope and, consequently, a similar change to TMp waveforms (as seen in Fig. 10). Manwaring et al. acquired pulsation of the supraorbital artery just above the eye from subjects on the tilt table using an oximeter^50^. Since this artery originates from intracranial space, changes in intracranial compliance are evident in its blood flow. When ICP was increased by tilting, changes in the blood flow pulse waveform and tympanic membrane pulsation waveform were found to be similar^50^, further supporting the results of the current study. Hyperventilation is associated with reduced partial pressure of arterial CO_2_ and decreases the subject’s ICP level^36^. Therefore, a hyperventilation-induced ICP decrease tended to reverse the change in TMp waveform slope ratio in all study subjects (P = 5.1E−6, paired two tailed t-test). However, the change in slope with hyperventilation did not lead to a complete reversal of the slope ratio (Fig. 10), possibly due to a partial decrease in the ICP that did not completely eliminate the ICP increase induced by head-down-tilting. And lastly, after subjects returned to the head-up-tilt, the TMp regained its initial shape displayed at the first head-up-tilt at the beginning of the experiment. This suggests a fast return to normal ICP levels in the current study subjects.

To investigate the dependence of the TMp signal quality on the measurement location, TMp signals were acquired from both ears and from the left and right ears separately to help choose the TMp waveform with the highest quality. Higher signal quality will likely help find possible TMp correlations with ICP changes. As can be seen in Table 1, the variations in the TMp signal quality from the right, left and both ears were mostly within 30% in 10 of the 15 study subjects. While the differences in the rest 5 subjects (subject 3, 4, 10, 11, 14) were as high as 90%. The signal quality differences may be due to unilateral factors, which may include tympanic membrane perforation, middle ear infections, and non-symmetric inhibition of the pressure transmission (i.e. through the cochlear aqueduct). An otoscope examination before TMp signal acquisition is recommended to assess the outer and middle ear conditions.

The inter- and intra-subject variability of the signal quality in the current study ranged from 0.11 to 0.43 (Table 1). Results also suggested that a signal quality value as low as 0.23 (subject 3, both ears) was sufficient to determine the parameters of interest (slope ratio and energy ratio) and detect the elevated ICP in the study subjects. In a clinical environment, the highest signal quality may be achieved by measuring the TMp signal in the right and left ear separately as well as in the both ears. Then the signal with the highest quality would be chosen for further experiment. However, if the signal quality was too low to properly calculate the parameters of interest, this may pose a use limitation of the proposed method. This situation may be encountered if the signal quality is significantly lower than 0.23. Scenarios when signal quality may be reduced include outer ear canal blockages, earwax impaction, middle ear infections. Possible method of improving the signal quality may include using more sensitive pressure transducer with lower background noise levels.

Limitations of the current study include selecting subjects with a relatively narrow age range. In addition, the patency of the cochlear aqueduct may decrease with age^28^, which may reduce the effectiveness of the proposed method in older patient populations. The study included only healthy subjects and the sample size was relatively small. Investigating a larger number of subjects and patients with different neurological disorders will help further generalize the results of the study. Alterations in ICP were induced by well-established physiological maneuvers, but head-down-tilt is a complex process that may not fully replicate pathological ICP elevation. ICP was not directly measured in the current study since the risks of invasive ICP measurements were not justified in the participating normal subjects. However, direct ICP measurement may be available in patients at risk of elevated ICP, especially in individuals whose ICP is already being monitored. Future studies may include a larger number of subjects and patients at risk of elevated ICP. In addition, a sensitive pressure transducer that can acquire TMp from the ear canal with short or no tubing may be more favorable in a clinical setting as it can reduce the noise induced by patient and tubing movements.

## Conclusion

This paper describes a new method for noninvasive detection of elevated ICP using tympanic membrane pulsation measurements. TMp waveforms were acquired in healthy subjects by a pressure sensor and showed consistent morphological changes with tilting subjects in the head-down position and with hyperventilation. Changes in waveform morphology were describable by a slope ratio calculation. The slope ratio was consistently larger in the head-down-tilt (increased ICP) position and decreased with hyperventilation (decreased ICP), (*p* < 0.01). When subjects returned to head-up-tilt, the TMp waveforms also returned to their initial morphology. The energy ratio in certain frequency bands was used to quantify the relative amplitude of high frequency peaks. The differences in the slope and energy ratio between normal and elevated ICP states may be used to distinguish between normal and elevated ICP states using thresholds, where separation accuracy was 100% and 76% respectively, in the current study. The study results suggest that using slope ratios to identify elevated ICP may be more valuable since it did not require availability of baseline measurements, which increases the potential utility of the proposed method.

The study results are encouraging as they may result in a relatively simple, noninvasive diagnostic technique to detect ICP elevation. More studies are needed to confirm the generality of these results in a larger number of subjects, including patients with (or at risk of) elevated ICP.

## References

[1] Dunn LT (2002). Raised intracranial pressure. J. Neurol. Neurosurg. Psychiatry.

[2] Daugherty J, Waltzman D, Sarmiento K, Xu L (2019). Traumatic brain injury-related deaths by race/ethnicity, sex, intent, and mechanism of injury - United States, 2000–2017. MMWR Morb. Mortal Wkly. Rep..

[3] Kristiansson H (2013). Measuring elevated intracranial pressure through noninvasive methods: A review of the literature. J. Neurosurg. Anesthesiol..

[4] Sullivan PG (2000). Dose-response curve and optimal dosing regimen of cyclosporin A after traumatic brain injury in rats. Neuroscience.

[5] Freeman WD (2015). Management of intracranial pressure. Continuum (Minneap Minn).

[6] Tavakoli S, Peitz G, Ares W, Hafeez S, Grandhi R (2017). Complications of invasive intracranial pressure monitoring devices in neurocritical care. Neurosurg. Focus.

[7] Khan MN, Shallwani H, Khan MU, Shamim MS (2017). Noninvasive monitoring intracranial pressure - A review of available modalities. Surg. Neurol. Int..

[8] Geeraerts T, Duranteau J, Benhamou D (2008). Ocular sonography in patients with raised intracranial pressure: The papilloedema revisited. Crit. Care.

[9] Alperin NJ, Lee SH, Loth F, Raksin PB, Lichtor T (2000). MR-Intracranial pressure (ICP): A method to measure intracranial elastance and pressure noninvasively by means of MR imaging: baboon and human study. Radiology.

[10] Klingelhöfer J, Conrad B, Benecke R, Sander D (1987). Intracranial flow patterns at increasing intracranial pressure. Klin. Wochenschr..

[11] Reid A (1990). The relationship between intracranial pressure and tympanic membrane displacement. Br. J. Audiol..

[12] Beentjes BI (1972). The cochlear aqueduct and the pressure of cerebrospinal and endolabyrinthine fluids. Acta Otolaryngol..

[13] Marchbanks, R.J. Hydromechanical interactions of the intracranial and intralabyrinthine fluids. in *Intracranial and Intralabyrinthine Fluids: Basic Aspects and Clinical Applications* (eds. Ernst, A., Marchbanks, R. & Samii, M.). 51–62 (Springer, 1996).

[14] Azad, M. K. *et al*. Monitoring intracranial pressure using non-invasive brain stethoscope. in *SoutheastCon 2018* (IEEE, 2018).

[15] Cardoso ER, Rowan JO, Galbraith S (1983). Analysis of the cerebrospinal fluid pulse wave in intracranial pressure. J. Neurosurg..

[16] Czosnyka M (2007). Intracranial pressure: More than a number. Neurosurg. Focus.

[17] Czosnyka Z (2008). Pulse amplitude of intracranial pressure waveform in hydrocephalus. Acta Neurochir. Suppl..

[18] Kirkness CJ, Mitchell PH, Burr RL, March KS, Newell DW (2000). Intracranial pressure waveform analysis: Clinical and research implications. J. Neurosci. Nurs..

[19] El-Bouri WK (2018). Quantifying the contribution of intracranial pressure and arterial blood pressure to spontaneous tympanic membrane displacement. Physiol. Meas..

[20] Gwer S (2013). The tympanic membrane displacement analyser for monitoring intracranial pressure in children. Childs Nerv. Syst..

[21] Samuel M, Burge DM, Marchbanks RJ (1998). Tympanic membrane displacement testing in regular assessment of intracranial pressure in eight children with shunted hydrocephalus. J. Neurosurg..

[22] Finch LC, Marchbanks RJ, Bulters D, Birch AA (2018). Refining non-invasive techniques to measure intracranial pressure: Comparing evoked and spontaneous tympanic membrane displacements. Physiol. Meas..

[23] Sharif SJ, Campbell-Bell CM, Bulters DO, Marchbanks RJ, Birch AA (2018). Does the variability of evoked tympanic membrane displacement data (V m) increase as the magnitude of the pulse amplitude increases. Acta Neurochir. Suppl..

[24] Shimbles S, Dodd C, Banister K, Mendelow AD, Chambers IR (2005). Clinical comparison of tympanic membrane displacement with invasive intracranial pressure measurements. Physiol. Meas..

[25] Dhar, R., Sandler, R. H., Manwaring, K. & Mansy, H. A. Noninvasive detection of elevated intracranial pressure using tympanic membrane pulse. in *2019 IEEE Signal Processing in Medicine and Biology Symposium* (SPMB) (IEEE, 2019).

[26] Dhar, R., Sandler, R. H., Manwaring, K. & Mansy, H. A. Spectral analysis of tympanic membrane pulse signal: An approach for noninvasive detection of elevated intracranial pressure. in *2020 IEEE Signal Processing in Medicine and Biology Symposium* (SPMB) (IEEE, 2019).

[27] Kostick N, Manwaring K, Dhar R, Sandler R, Mansy H (2021). The, "brain stethoscope": A non-invasive method for detecting elevated intracranial pressure. Cureus.

[28] Włodyka J (1978). Studies on cochlear aqueduct patency. Ann. Otol. Rhinol. Laryngol..

[29] *LabScribe Data Acquisition and Analysis Software, version 3.611700*. (iWorx Systems Inc., 2021). https://iworx.com/labscribe/.

[30] *MATLAB and Statistics Toolbox Release 2018a*. (The MathWorks Inc., 2021). https://www.mathworks.com/products/matlab.html.

[31] Evensen KB, Paulat K, Prieur F, Holm S, Eide PK (2018). Utility of the tympanic membrane pressure waveform for non-invasive estimation of the intracranial pressure waveform. Sci. Rep..

[32] Gopen Q, Rosowski JJ, Merchant SN (1997). Anatomy of the normal human cochlear aqueduct with functional implications. Hear Res..

[33] Taebi A, Mansy HA (2016). Effect of noise on time-frequency analysis of vibrocardiographic signals. J. Bioeng. Biomed. Sci..

[34] Westhout FD, Paré LS, Delfino RJ, Cramer SC (2008). Slope of the intracranial pressure waveform after traumatic brain injury. Surg. Neurol..

[35] Balestreri M (2004). Intracranial hypertension: what additional information can be derived from ICP waveform after head injury. Acta Neurochir (Wien).

[36] Morgalla MH, Stumm F, Hesse G (1999). A computer-based method for continuous single pulse analysis of intracranial pressure waves. J. Neurol. Sci..

[37] Holm S, Eide PK (2008). The frequency domain versus time domain methods for processing of intracranial pressure (ICP) signals. Med. Eng. Phys..

[38] Eide PK, Brean A (2006). Intracranial pulse pressure amplitude levels determined during preoperative assessment of subjects with possible idiopathic normal pressure hydrocephalus. Acta Neurochir (Wien).

[39] Eide PK, Sorteberg W (2006). Intracranial pressure levels and single wave amplitudes, Glasgow Coma Score and Glasgow Outcome Score after subarachnoid haemorrhage. Acta Neurochir (Wien).

[40] Hargens AR, Hargens AR (1994). Recent bed rest results and countermeasure development at NASA. Acta Physiol. Scand. Suppl..

[41] Tatebayashi K (2003). Effects of head-down tilt on the intracranial pressure in conscious rabbits. Brain Res..

[42] Raichle ME, Plum F (1972). Hyperventilation and cerebral blood flow. Stroke.

[43] Rodríguez-Boto G, Rivero-Garvía M, Gutiérrez-González R, Márquez-Rivas J (2015). Basic concepts about brain pathophysiology and intracranial pressure monitoring. Neurologia.

[44] Nag DS, Sahu S, Swain A, Kant S (2019). Intracranial pressure monitoring: Gold standard and recent innovations. World J. Clin. Cases.

[45] Carrera E (2010). What shapes pulse amplitude of intracranial pressure. J. Neurotrauma.

[46] Stettin E, Paulat K, Schulz C, Kunz U, Mauer UM (2011). Noninvasive intracranial pressure measurement using infrasonic emissions from the tympanic membrane. J. Clin. Monit. Comput..

[47] Hommerich KW (1964). Hearing disorders and disturbances of the olfactory system in intracranial diseases. Arch. Ohren Nasen Kehlkopfheilkd.

[48] Arani A (2018). Acute pressure changes in the brain are correlated with MR elastography stiffness measurements: initial feasibility in an in vivo large animal model. Magn. Reson. Med..

[49] Kawai Y (2003). Effects of microgravity on cerebral hemodynamics. Yonago Acta Med..

[50] Manwaring, P., Manwaring, J., Manwaring, K., & Manwaring, M. A provocative test to determine brain compliance in the management of patients with hydrocephalus. in *2005 IEEE Engineering in Medicine and Biology 27th Annual Conference* (IEEE, 2005). 10.1109/IEMBS.2005.1616037 17281806

